# Tear and blood salusin-α, and -β, copeptin, and asprosin in patients with glaucoma and ocular hypertension

**DOI:** 10.5935/0004-2749.2024-0230

**Published:** 2025-06-24

**Authors:** Sermal Arslan, Mehmet Kaan Kaya, Süleyman Aydin

**Affiliations:** 1 Universaleye Clinic, Elazig, Turkiye; 2 Department of Medical Biochemistry and Clinical Biochemistry, (Firat Hormones Research Group), Medical School, Firat University, Elazig, Turkiye

**Keywords:** Glaucoma, open-angle/physiopathology, Intraocular pressure/physiopathology, Retinal ganglion cells/pathology, Biomarkers/blood, Glycopeptides, Fibrillin-1, Tears/chemistry, Intercellular signaling peptides and proteins/blood, Enzyme--linked immunosorbent assay/methods

## Abstract

**Purpose:**

This pilot study was conducted to investigate the presence of various bioactive compounds (copeptin, asprosin, and salusins) in the blood and tears of patients with glaucoma.

**Methods:**

A total of 83 subjects, including 28 patients with open-angle glaucoma, 28 patients with ocular hypertension, and 27 control volunteers, were enrolled in this study. The levels of salusin-α, salusin-β, copeptin, and asprosin in tears and venous blood samples were measured by enzyme linked immunosorbent assay (ELISA).

**Results:**

Patients with open-angle glaucoma and those with ocular hypertension showed statistically significantly decreased levels of salusin-α and salusin-β in their blood and tears compared with those of control subjects (p<0.05), with the decrease being the most pronounced in patients with ocular hypertension (p<0.05). In contrast, the levels of copeptin and asprosin showed a statistically significant increase in both these patient groups compared with those of control subjects (p<0.05). There was a negative correlation between intraocular pressure and blood and tear salusins.

**Conclusions:**

Fluids from patients with open-angle glaucoma and ocular hypertension showed lower salusin levels. Patients with ocular hypertension had higher levels of copeptin and asprosin, but not those with open-angle glaucoma (except for asprosin, whose levels showed a slight but remarkable increase in plasma in patients with open-angle glaucoma). The pathogenesis of ocular hypertension and open-angle glaucoma may be significantly impacted by these biomarkers.

## INTRODUCTION

A class of ocular neuropathies known as glaucoma is distinguished by the gradual degradation of retinal ganglion cells. In these diseases, degeneration of the nerves of the central nervous system, whose cell bodies are located in the inner retina and whose axons are located in the optic nerve, results in crusting characteristics of the optic nerve head and the consequent loss of vision^(1)^. Irreversible vision loss results from a delayed diagnosis of glaucoma because this biological cascade of events is typically asymptomatic. Approximately 10% of people worldwide have bilateral blindness, and more than 76 million have glaucoma^(2)^. Low-pressure glaucoma, or open-angle glaucoma with normal intraocular pressure, is prevalent throughout the world. Ocular hypertension (OHT) and glaucoma may occur as clinical complications of ocular surface inflammatory diseases (e.g., atopic keratoconjunctivitis). In open-angle glaucoma, visual field deterioration and optic nerve impairment emerge despite normal intraocular pressure (IOP)^(1^,^3)^. In this type of glaucoma, although the intraocular pressure does not exceed 22 mmHg, the eyes become vulnerable even with normal intraocular pressure (IOP) due to sensitization and weakening of the optic nerve head caused by impaired blood flow. In other words, when the intraocular pressure is normal, the optic disk becomes hollow and visual field loss occurs. Conversely, no visual field loss or optic nerve damage is observed in OHT despite the high intraocular pressure. If the intraocular pressure is ≥22 mmHg with no visible damage to the optic nerve fibers, this condition is not classified as glaucoma. Nevertheless, close monitoring is required because it may develop into glaucoma. The prevalence of OHT in the general population ranges between 2.7% and 3.8%^(4^,^5)^.

Salusins are vasoactive peptides consisting of 28 and 20 amino acids as salusin-α and salusin-β, respectively^(6)^. They are present in biological tissues of the kidney, central nervous system, and vascular system^(7)^. Salusin-α acts as an antiatherogenic factor, whereas salusin-β physiologically acts as a proatherogenic factor. The release of salusin-β is stimulated by inflammatory cytokines such as tumor necrosis factor alpha (TNF-α) and lipopolysaccharides^(8)^. Moreover, salusin--β increases the infiltration of macrophages^(9)^. Furthermore, microinjection of salusin-β into the paraventricular nucleus increases blood pressure by releasing norepinephrine and arginine vasopressin^(10)^. Salusin-α has also been reported to exert an antiatherogenic effect by reducing the formation of macrophage foam cells and the expression of cholesterol acyltransferase 1 (ACAT-1)^(11)^.

Copeptin, the C-terminal peptide of provasopressin, is a glycopeptide molecule composed of 39 amino acids (rich in leucine). It is released from the neurohypophysis and reflects arginine vasopressin hormone activity in patients with hypertension^(12)^. Although vasopressin (released with copeptin in a stoichiometric ratio, i.e., equimolar 1:1) is primarily involved in regulating water balance and electrolyte homeostasis, it also regulates blood pressure. Elevated circulating levels of copeptin have been associated with cardiovascular disease (particularly hypertension and heart failure) and mortality^(13)^. Therefore, copeptin may be involved in the pathophysiology of glaucoma. Nevertheless, there is yet no research on this topic (regarding glaucoma).

Asprosin is a product of the fibrillin-1 (*FBN1*) gene and a C-terminal cleavage product of profibrillin, which consists of 140 amino acids and controls hepatic glucose excretion. The name asprosin (ASP) is derived from the Greek word “aspros,” meaning “white,” because it was first founded to be secreted by white adipose tissue^(14)^. The best known physiological effect of ASP is inducing rapid glucose release from the liver. High plasma levels of asprosin may play a protective role in cardiovascular events [e.g., in patients with dilated cardiomyopathy (DCM)], and its underlying protective mechanisms may be related to improved mitochondrial respiratory functions under hypoxia^(15)^. Long-term studies have demonstrated that patients with glaucoma have altered blood flow measurements^(16^,^17)^. Hence, endothelial dysfunction and narrowing of blood vessels will increase the resistance to flow distally, thereby causing distal tissue hypoxia^(18)^.

Because ASP may play a protective role in hypoxia^(15)^, it is worth exploring whether it also functions in patients with glaucoma, because a small change in the supply of eye vessels will result in vascular endothelial dysfunction^(18)^.

The above-described overview indicates that the pathophysiological mechanisms of glaucoma and the factors affecting its progression remain inadequately understood. Therefore, the objectives of this study were to clarify the characteristics of the molecules salusin-α, salusin-β, copeptin, and asprosin in concurrently collected tears and blood from patients with low-tension glaucoma and OHT, as well as from healthy individuals, and to determine the potential association of these molecules with the pathophysiology of these ocular conditions.

## METHODS

This study was conducted at the Universal Eye Hospital and the Faculty of Medicine of Firat University with approval obtained from the Noninterventional Ethics Committee of Firat University dated January 12, 2023, and numbered 20233/01-18. Participants were informed about the study, and informed consent was obtained from each of them. The study was conducted according to the ethical standards of the 1983 revision of the Declaration of Helsinki. In total, 28 patients with open-angle glaucoma (intraocular pressure 21-22 mmHg), 28 patients with OHT (intraocular pressure 29-30 mmHg), and 27 volunteers (control group) with normal intraocular pressure (16 mmHg) who were suspected to have open-angle glaucoma but were determined to have no health or eye problems were included. A medical history was obtained, and a physical examination was performed on these participants. Selected patients had OHT and open-angle glaucoma, both of which were newly diagnosed and untreated. Patients with preexisting chronic obstructive pulmonary disease and liver disease, those with acute myocardial infarction, patients with cataract and retinopathy, patients with diabetes mellitus, those with renal failure, patients with a history of hypothyroidism or hyperthyroidism and cardiac cachexia, morbidly obese patients, patients aged <18 and >80 years, and those with active infections and a history of cerebrovascular disease were excluded. The body mass index (BMI) of the subjects was calculated by dividing weight in kilograms by height in square meters. From all participants, 10 µL of tear samples were collected into Eppendorf tubes using nonirritant capillary pipettes without anesthesia, as described previously^(19)^, along with 5 mL of venous blood, as described earlier^(20)^. These samples were centrifuged at 4000 rpm for 5 min and stored in Eppendorf tubes at -40°C until being assayed^(21)^. This method was followed to collect tear fluid at a volume that would enable additional laboratory analysis in a manner that would be practical for the patient and applicable to clinical routine^(19)^; moreover, this method can be easily reproduced by other researchers^(19)^. These techniques are also easy to use and inexpensive.

### Examination of blood and tear samples by ELISA

The levels of human salusin-α (catalog no: 201-12-1269), salusin-β (catalog no: 201-12-1273), copeptin (catalog no: 201-12-5463), and asprosin (catalog no: 201-12-7193) *were measured by ELISA using commercially available ELISA kits obtained from* Sunred Biological Technology Co., Ltd, Shanghai, China, according to the kit procedure. The intra-assay coefficient of variation (CV) (change within the day) of all kits used in this study was <10%, and the interassay CV (between days) was <12%. Assay validation for tear samples was performed as described previously^(22)^. The Bio-Tek ELX50 automated washing machine (BioTek Instruments, USA) was used for plate washes. Absorbance values *were measured using the ChroMate Microplate Reader P4300 (Awareness Technology Instruments, USA) at a wavelength of 450 nm.* The biochemical parameters (triglycerides, HDL cholesterol, LDL cholesterol, VLDL cholesterol, and glucose) in patient serum were measured using an autoanalyzer.

### Statistical analysis

All statistical analyses were conducted using computer programs (SSPS-22). In the analysis of study data, in addition to descriptive statistical methods, standard deviation (SD), Student’s t-test in parametric tests with normal distribution when comparing quantitative data, one-way analysis of variance (one-way ANOVA) in comparisons between groups and significance test of the difference between two pairs Wilcoxon paired two--sample test and the results were evaluated at the 95% confidence interval, a significance level of p<0.05.

## RESULTS

No statistically significant difference was found between the participants’ age, BMI, and blood glucose levels (Table 1). A detailed summary of the demographic data, clinical symptoms, and indicators of the study participants is provided in Table 1. The right and left eye pressures of patients with open-angle glaucoma (OAG) and those with OHT were statistically significantly higher than the right and left eye pressures of control subjects, respectively (p<0.05) (Table 1).

**Table 1:** Demographic information, clinical symptoms, and signs of the study participants

Demographics	Control (n=27)	OAG (n=28)	OH (n=28)
**Female (n)**	13	12	13
**Male (n)**	14	16	15
**Age (y), mean ±SD**	75 ± 5	74 ± 6	72 ± 9
**BMI (kg/m** ^^2^^ **), mean± SD**	24 ± 4.2	25 ± 4.6	26 ± 5.6
**Glucose (mg/dL)**	98 ± 4.2	108 ± 5.6	101 ± 4.2
**TG (mg/dL)**	118 ± 8.6	124 ± 9.5	119 ± 6.8
**LDL-C (mg/dL)**	112 ± 9.3	128 ± 9.8	133 ± 11.6
**HDL-C (mg/dL)**	47.8 ± 3.8	43.6 ± 3.2	42.8 ± 2.9
**Systolic blood pressure (mm Hg)**	127 ± 4.8	132 ± 5.1	138 ± 7.8 ^a^
**Diastolic blood pressure (mm Hg)**	84 ± 2.9	81 ± 3.4	86 ± 7.2
**Right eye pressure (mmHg)**	16.6 ± 2.6	21.32 ± 2.9	29.45 ± 3.2 ^a^
**Left eye pressure (mmHg)**	15.9 ± 2.9	22.52 ± 3.1	30.7 ± 4.5 ^a^

BMI= body mass index; TG= triglyceride, LDL-C= low-density lipoprotein cholesterol; HDL-C= high-density lipoprotein cholesterol; a= Control group versus OHT (p<0.05); a= Control right eye pressure versus right eye pressure with OHT (p<0.05); a= Control left eye pressure versus left eye pressure with OHT (p<0.05).

a: Blood control group versus blood ocular hypertension group (p<0.05).

b: Tear control group versus tear ocular hypertension group (p<0.05).

The tear ELISA kits utilized in the study were found to quantify this molecule precisely based on the results of their validation testing. The CV for the intra-assay (change within a single day) was <10%, and the CVs for the interassay (change between days) were <12% and 14%. The results were linear, and no molecules were lost during recovery. Table 2 provides a summary of results of all the experimental validity tests.

**Table 2:** Intra-assay and interassay values in the tear fluid determined using ELISA kits

Molecules	Min/Max	Intra (CV %)	Inter (CV %)	Recovery	Linearity
**Salusin-α**	0.05/5250 (pg/mL)	<10	<12	102%	Yes
**Salusin-β**	0.05/5500 (pg/mL)	<10	<12	93%	Yes
**Copeptin**	0.07/20 (ng/mL)	<8	<14	96%	Yes
**Asprosin**	0.01/20 (ng/mL)	<8	<12	116%	Yes

a: Blood control group versus blood open-angle glaucoma group (p<0.05).

b: Blood control group versus blood ocular hypertension group (p<0.05).

c: Blood open-angle glaucoma group versus blood ocular hypertension group (p<0.05).

d: Blood control group versus tear control group (p<0.05).

e: Tear control group versus tear ocular hypertension group (p<0.05).

**Figure 1:**
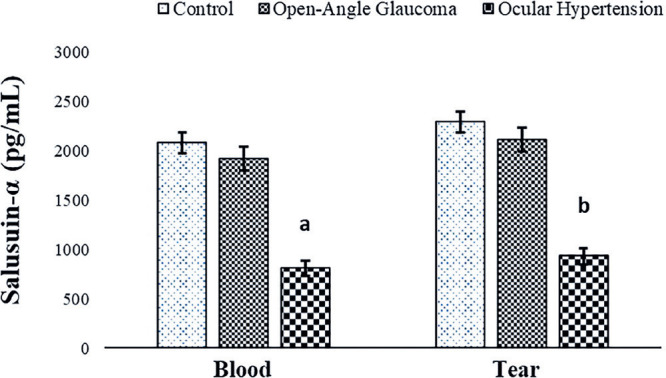
Comparison of salusin-α levels in the blood and tear fluid of patients with open-angle glaucoma and those with ocular hypertension (OHT) and control subjects.

To our knowledge, salusin-α and -β molecules were detected for the first time in the blood and tears of patients with OHT and open-angle glaucoma, as well as in control subjects. Salusin-α levels showed a decrease in the blood and tears of the patient groups compared with those of the control group, with the decrease being statistically significant in patients with OHT (p<0.05) (Figure 1).

**Figure 2:**
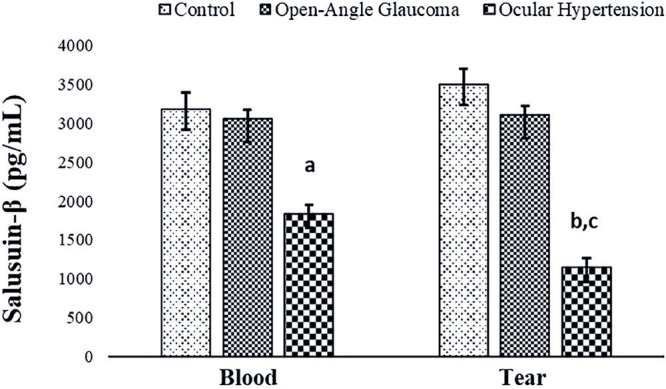
Comparison of salusin-β levels in the blood and tear fluid of patients with open-angle glaucoma and those with ocular hypertension (OHT) and control subjects.

Salusin-β levels also showed a similar trend, with statistically significantly lower levels in patients with OHT (p<0.05) than in patients with open-angle glaucoma (Figure 2). However, blood and tear salusin-α and -β levels showed no statistically significant differences.

a: Blood control group versus blood ocular hypertension group (p<0.05).

b: Tear control group versus tear ocular hypertension group (p<0.05).

**Figure 3:**
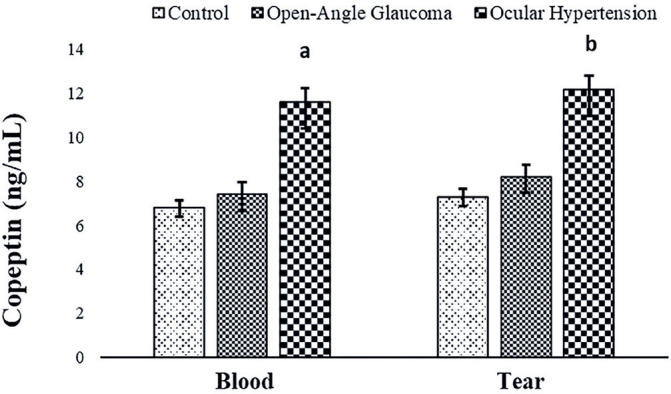
Comparison of copeptin levels in the blood and tear fluid of patients with open-angle glaucoma and those with ocular hypertension (OHT) and control subjects.

**Figure 4:**
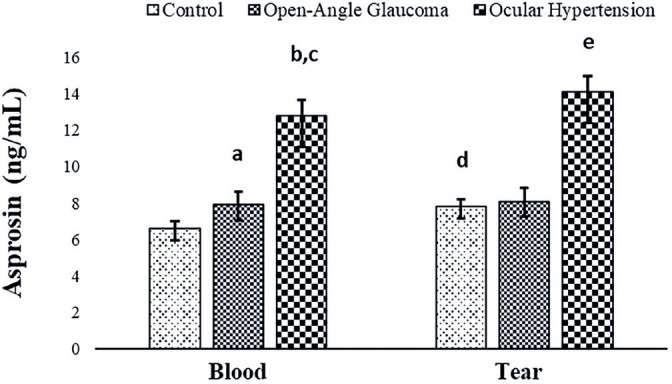
Comparison of asprosin levels in the blood and tears of patients with open-angle glaucoma and those with ocular hypertension (OHT) and control subjects.

Copeptin and asprosin molecules were quantified for the first time in this study in the blood and tears of patients with open-angle glaucoma and those with OHT, as well as in control subjects (Figures 3 and 4).

a: Blood control group versus blood ocular hypertension group (p<0.05).

b: Tear control group versus tear ocular hypertension group (p<0.05).

c: Blood ocular hypertension group versus tear ocular hypertension group (p<0.05).

In contrast to salusin levels, copeptin and asprosin levels were increased in the blood and tears of the patient groups compared with those in the control group, with the increase being statistically significant in the OHT group (p<0.05) compared with copeptin levels in the blood and tears of the open-angle glaucoma and OHT groups (Figure 3). Asprosin levels in the blood and tears of patients with OHT and those with open-angle glaucoma were considerably higher (p<0.05) than those in the control group, with the levels being statistically significantly higher in patients with OHT than in patients with open-angle glaucoma (Figure 4).

With intraocular blood pressures (for salusin-α r=-0.577, p<0.05; for salusin-β r=-0.483, p<0.05) and tear salusins (for salusin-α r=-0.494, p<0.05; for salusin-β r=-0.612, p<0.05) there was a negative correlation (r=0.616, p<0.05) and there were positive correlations between tears (r=0.422, p<0.05) copeptin and asprosin (blood r=0.514, p<0.05; tears r=0.478, p<0.05).

## DISCUSSION

To our knowledge, the levels of salusin-α and -β, asprosin, and copeptin in the blood and tears of patients with open-angle glaucoma and those with OHT were investigated for the first time in this study. Although patients with OHT and those with open-angle glaucoma had lower blood and tear salusin-α and -β levels than control subjects, these decreases were primarily observed in patients with OHT. Patients with a history of coronary artery disease, ischemic heart disease, hypertension, and chronic renal failure have also been found to have lower blood levels of salusin-α and -β^(11^,^22^,^23)^. Salusins were initially identified by Shichiri et al. in 2003^(6)^ and are produced in organs such as human vascular endothelial cells, kidneys, and brains^(24)^.

Moreover, patients with mild hypertension have significantly lower levels of salusin-α^(25)^. Salusin levels decrease in patients with OHT, similar to those in patients with open-angle glaucoma, moderate hypertension, and OHT. Therefore, the pressure inside the eye increases, and the retinal nerve fibers are mechanically destroyed^(26)^. Hence, the regulation of intraocular pressure and aberrant resistance in the trabecular meshwork may be linked to the decrease of salusin levels under these conditions. Evaluating salusin levels in patients with open-angle glaucoma and those with OHT can help stop vision loss before it occurs entirely because both conditions advance subtly and without visible signs.

Copeptin levels in the blood and tears of patients with open-angle glaucoma and those with OHT were also compared in this study, which revealed that, unlike salusin levels, copeptin levels increased significantly in patients with OHT and those with open-angle glaucoma compared with the levels in control subjects, with the increase being the maximum in patients with OHT. Moreover, copeptin levels in the blood and tears of patients with open-angle glaucoma were significantly higher than those of control subjects. Studies have reported higher levels of copeptin in patients with hypertension or conditions linked to hypertension, such as a previous joint illness, than in control subjects^(12^,^27^,^28)^. Copeptin has also been investigated as a possible biomarker for the prognosis and diagnosis of numerous other illnesses, including preeclampsia. According to Wang et al.^(29)^, copeptin may be a novel unique biomarker for preeclampsia We found that copeptin levels in blood, tears, and blood pressure correlated positively. These findings suggest that copeptin is involved in differentiating between OHT and open-angle glaucoma, which is due to the fact that elevated copeptin levels in blood and tears were more closely linked to OHT than to open-angle glaucoma.

Similar to copeptin levels, asprosin levels in the blood and tears of patients with OHT and those with open-angle glaucoma were significantly higher than control levels in both conditions, with the largest increa-ses detected in patients with OHT. A previous study reported that serum asprosin levels correlated positively with diastolic blood pressure^(30)^. To our knowledge, this study is the first to identify a positive correlation between asprosin levels in the tears and blood of patients with open-angle glaucoma and those with OHT and their blood pressure. Elevated asprosin levels in tears and blood can provide physicians clues regarding the progression of OHT and open-angle glaucoma. This is because the increase in asprosin levels in tears and blood was greater in patients with OHT than patients with open-angle glaucoma. Nevertheless, the molecular mechanisms and functions of asprosin in diseases still remain unclear.

This study measured salusin-α and -β, copeptin, and asprosin levels in tears for the first time. According to the assay validity results, the ELISA kits used in this study measured these molecules with the same sensitivity as in serum and plasma. Although commercial ELISA kits are manufactured to measure analytes in plasma or serum, companies indicate in their catalogs that the kits used in research laboratories (as they are not intended for diagnostic purposes) can also measure biological fluids other than blood. Nevertheless, similar to this study, it is useful to perform assay validity experiments according to previously published methods to determine the accuracy and sensitivity of these kits^(22)^.

This study is subject to certain limitations. The primary weakness is the limited number of participants. The relatively small sample size may affect both the statistical power and generalizability of findings. Eye drop therapy and intraocular pressure may exert an effect on biomarker levels. Moreover, a significant limitation is the absence of long-term follow-up data, which is essential to completely comprehend the prognostic significance of these biomarkers. Future studies could significantly improve our understanding of these crucial situations by addressing these limitations.

In conclusion, this study demonstrated that the concentrations of salusin-α and -β in the blood and tears of patients with open-angle glaucoma and those with OHT were lower. OHT was associated with higher levels of copeptin and asprosin. Furthermore, the results of the tear and blood concentrations of these molecules were similar. This study may provide a comprehensive view that could pave the way for future research.
